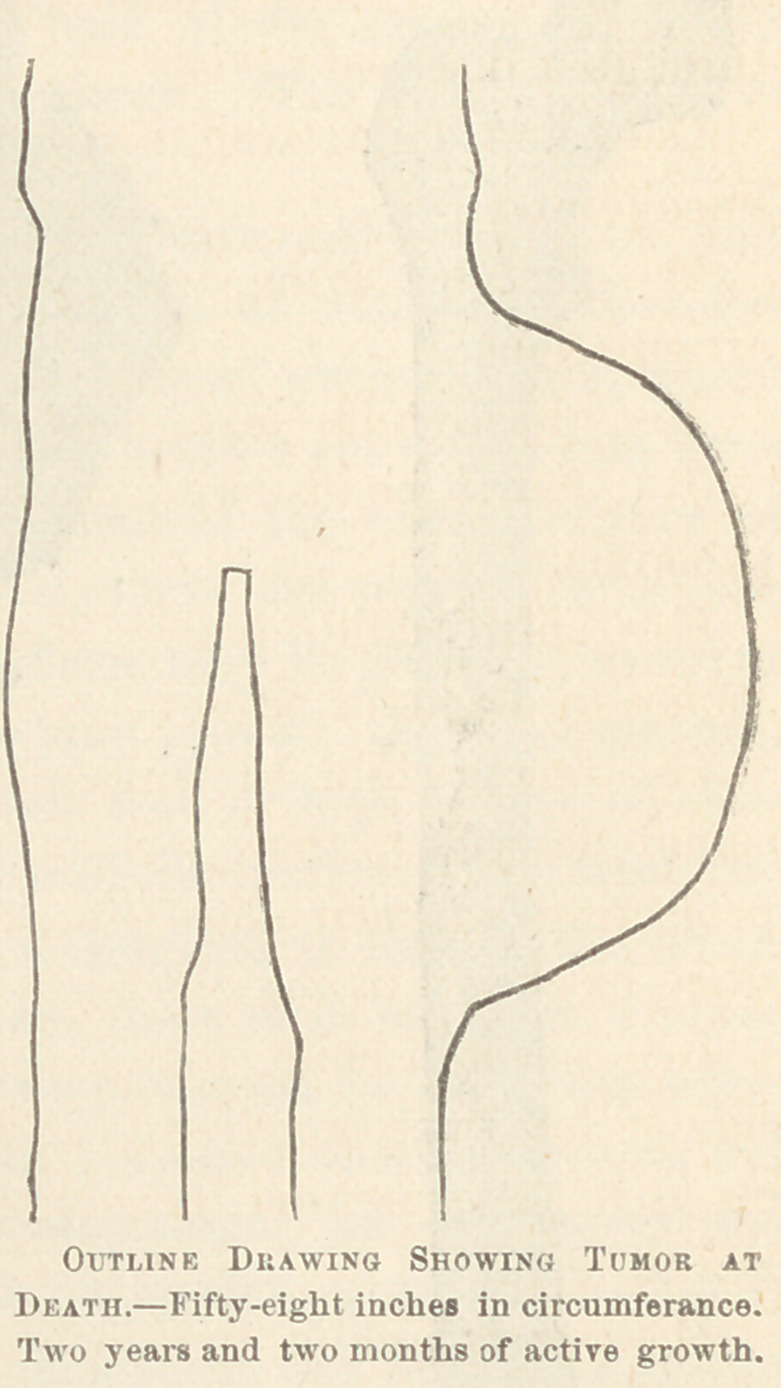# Osteocephaloma of the Thigh

**Published:** 1880-02

**Authors:** J. G. Meacham

**Affiliations:** Racine


### Selections.

OSTEOCEPHALOMA OF THE THIGH. Report to the Wisconsin State Medical Society. By J. G. MEACHAM, JR., M. D., of Racine.

On the tenth day of March, 1877, George G———, a young man of about twenty years of age, of German descent, came to my office to consult me about a tumor of his left thigh. He stated that for several years there had been on the outer side of the thigh a flat bunch, oval in outline, about two inches in diameter, which up to six months previous had given him no trouble, and had shown no signs of growth, but at that time, September, 1876, after violent exercise by jumping, it began to grow and had continued its development until the present time.

An examination of the tumor located it on the outer side of the left thigh, occupying the middle two-fourths in length, and firmly adherent to the bone. Its transverse diameter was greater than its anterio-posterior diameter, thereby presenting a broad flattened aspect. Its upper half was harder and less elastic than the lower part, and its general form was globular. The surface was somewhat lobulated, purple in color, smooth and glossy, having numerous tortuous veins ramifying over it. The thigh was not at all increased in size along its inner line, and but slightly enlarged in front or behind, but outward the growth stood prominent and bold.

A measurement around its largest part at the middle of the thigh gave twenty-five inches; two inches below this it measured twenty-two inches, while two inches lower still it was only fifteen inches, the natural circumference of his thigh. Some, though not severe, darting pains were felt in the tumor, but it was complained of most as an encumbrance by its size and weight.

The physical powers of the body at this time were somewhat enfeebled, and there was emaciation, notwithstanding the subject was always thin and spare, weighing but one hundred and nineteen pounds when in health. The countenance was pale and expressive of anxiety and suffering, evidencing cachexia. The patient, however, regularly followed his usual occupation, that of engineer, and walked nearly a mile to and from the shop in which he was employed.

The one thought in my mind regarding this large and rapidly growing tumor was, that it was osteo-cancer, already beyond the reach of human aid. M. Amedée Forget, a French surgeon, in speaking of malignant osseous growths, asks this question: "Are there not, in the present state of science, clinical characters indicating to the surgeon whether or not to operate?" In this case, it seemed to me that the rapid growth, the peculiar form, the pain, the evident cachexia and emaciation, and the unfavorable location, were clinical characters enough to indicate the best thing to do, namely, let it alone. It was too large to be removed in any way, except, perhaps, by amputation at the hip-joint; but, considering the very great danger to life attending that operation, together with the probable infiltration of cancer-cells in the adjoining parts, it seemed best and consistent with good judgment, to discourage surgical interference and experiment, and to advise him to enjoy, under palliative means, his remaining months or years as best he could.

About four weeks subsequent to this he called again. There was no special change of growth in size or appearance. My father, Dr. J. G. Meachem, was present, and concurred in the diagnosis and advice I had previously given. Photographs of the tumor, made at that time, I now show you.*

Eight months had passed when the patient again came under observation at the office. During the time he had been subjected to all kinds of treatment by the usual number of medical pretenders that follow up a lingering, incurable case. His condition was one of greater weakness than before, with much more marked emaciation; the skin was of the hue peculiar to cancer cachexia, and the suffering was more acute and severe. The growth had

* Photographs were exhibited showing anterior and posterior views.

extended below nearly to the knee, and above to the hip, but did not yet interfere with motion in either joint. It also had overlapped the thigh anteriorly and posteriorly to its inner line. It now measured thirty-six and one-half inches in circumference at its middle, and thirty-two inches around two inches lower down, having, therefore, increased eleven and one-half inches, and this increase relatively the same throughout the entire mass. An appearance of still greater size was manifest in contrast to the greater emaciation of surrounding parts. A second photograph was made, and by comparing one with the other, the real increase is plainly shown. Up to this time he had followed his work, but now was obliged to remain at home, its bulk preventing locomotion.

OSTEOCEPHALOMA.—Anterior and posterior views when the tumor measured forty inches in circumference.—From photographs.

During the next two months it increased to forty inches. His weight at this time was one hundred and eighty-nine pounds. From this on for six months the usual condition of progressing emaciation and weakness continued, while the morbid growth enlarged day by day until it reached a size measuring fifty-eight inches (*nearly five feet*) in circumference. It now obliterated every natural outline of the thigh, hanging down over the knee, and encroaching on the abdomen above. The relative shape was retained throughout its development. The skin was still intact, though very thin, and from the peculiar velvety feel in places, I think ulceration would have occurred soon. There was also evidence of deep fluctuation.

I believe pus is seldom if ever found in malignant growths, for the simple reason that the action is not inflammatory but rather a process by which the cells assume such a property of self-multiplication that their normal relation and mode of arrangement is destroyed. (Bennett.)

Dr. F. H. Hamilton says that portions of the tumor have usually an elastic feel, leading often to the suspicion that it contains pus. The word "suspicion," I take it, refers to a supposed existence of something that does not exist, and that in these growths pus will not be found. Not unfrequently, however, small cavities filled with clotted blood, dirty-looking serum, or soft, gelatinous matter are formed within them (Gross); and a knowledge of this, together with a consideration of the vitality of the patient, caused me to refuse a demand to puncture the mass, and to insist that it be allowed to take its own course.

A friend of the young man called another practitioner to see the case, who at once plunged a trocar deep into the softened portion, giving escape to about six quarts of dark-colored watery fluid. There was but little change in shape on the escape of this fluid. The water of decomposition and the serum of the blood dripped constantly from the opening, draining his life away. Air entered the cavity, hastening decomposition; gases formed rapidly, and it was amid a stench unbearable that death came to his relief on the fourth day. I was not present when the puncture was made, but from the young man's mother I ascertained the amount of fluid that escaped at the first gush.

Twenty-four hours after death I was sent for to incise the tumor (which I did), to facilitate the placing of the body in the largest size coffin made. Circumstances and conditions were such on that day that no satisfactory post-mortem examination could be obtained. The day following the interment it came about that the relatives of the deceased should exhume the body and grant me permission to remove and have the tumor and thigh-bone. The body was exhumed, and I disarticulated the thigh.

The cavity seemed hardly large enough to have contained the quantity of fluid said to have escaped when tapped. It still held a little dark grumous fluid, such as results from disintegration of tissue; also some soft, white, brain-like matter, but no pus was to be seen. Throughout the entire mass were numerous pieces of cancellated bone-structure that had been formed in the progress of the disease. They radiated in every direction, uniting themselves one with another, forming a framework to the entire growth. The interspaces were gelatinous and brain-like, only a limited amount of cartilage being observed, and that in the upper part. The muscular structure was past recognition. The soft parts were under rapid decomposition, and it became necessary to hasten maceration.

The femur, as cleaned, shows that the disease involved nearly the entire bone; a small portion of the lower end, and the articular cartilage of the head of the bone, alone being exempt. This latter corresponds to a fact pointed out long ago by Petit, "that although the epiphysis may have been completely converted into encephaloid matter, the cartilage of incrustation and of the neighboring joint never becomes implicated, and this, although the growth may eventually involve and include the whole of the rest of the articulation by extension to the capsule and the soft parts." (Erichsen.)

The shaft at its middle was bent and twisted upon itself about forty-five degrees, probably by the great weight of the growth dragging it outward and backward. Radiating spicula stood out in all directions from the entire surface of the shaft. There were also pillar-like projections reaching outward to the cancellated formations, bracing and supporting all. The greatest density of bone was behind and below the trochanter major, which, corresponding to the point first complained of, would seem to locate the original site of disease, from thence extending down along the shaft. Morbid action undoubtedly began beneath the periosteum. During maceration, large numbers of pieces of cancellated bone separated from the femur, and I present them here with the femur, for your inspection to-day.* Portions of some of them

* Pieces of all sizes, sufficient to fill a half-peck measure.

have become compact bone structures, and several others have calcareous deposits upon their surfaces.

The lowest two inches of the femur is much broader and thicker than natural, not wholly from the disease, I think, but from a peculiar development belonging to a heredity that exists in the family, about which mention will be made further on.

A photograph of the bone, made to a scale of one-fourth inch to the inch, shows the enormous bulk of the osseous structure belonging to this pathological specimen. The comparative size is seen by the natural femur at its side.

The nature of the growth was unquestionably cancerous, and known as osteocephaloma. It could in no wise be confounded with any other growth, except that form of enchondroma, where portions of the cartilaginous element become converted into bone and when by a certain softening, some of the characteristics of soft cancer are presented. Indeed, there are several conditions common to both. The shape or form may be globular, ovoid or lobulated in both. The surface may present the same smooth, tense, glossy appearance. The soft feel, or high degree of elasticity in either, may convey a sense of fluctuation. The tendency to take on rapid growth in one, is kindred to that of the other. But in addition to the similarities, there is in our case sharper pain, which is a very marked accompaniment in cancerous affections; an unusually rapid growth (two and one-half years), not so common in cartilaginous developments; an emaciation and cachexy peculiar to cancer; and on section a small amount of cartilage, and a great amount of gelatinous and brain-like matter found, all giving greater probability of the presence of encephaloid disease. If the presence of fluid within the growth suggests to the minds of any of you cystic disease or cystoma, I would say simply that there was no membranous or osseous envelope, without which there could be no cyst. Central softening of the mass gave opportunity for the accumulation of fluid, under the same laws that govern the passive accumulation of fluids in the natural serous cavities of the body when there is existing neighboring disease.

The remarkable size of the morbid growth I would specially notice. Upon this point I have taken pains to consult such authorities as have been at my command, namely: Mott's Velpeau, Druitt, Erichsen, Gross, Hamilton, Bennett, Holmes, and Agnew, and about one hundred volumes of different medical journals. Most of these authors use expressions wholly indefinite, such as "considerable size," "large bulk," "great magnitude," "extraordinary development," etc., etc. A cocoanut and child's head are

OSTEOCEPHALOMA OF LEFT FEMUR.—A few of the pieces that separated during maceration shown. A natural femur to show contrast. From photograph.

instanced as representing the size of large osseous tumors. In the CHICAGO MEDICAL JOURNAL AND EXAMINER for December, 1877, is a synopsis of a case of malignant osseous tumor of the femur that measured twenty-five inches in circumference. In Dr.

Druitt's Surgery, edition of 1867, mention is made of a case of enchondroma by Dr. Frogley that reached a circumference of three feet, and another by Dr. Paget that was as large as a man's chest. These examples of large tumors are far short of the size of the one under consideration, and I am bold to say, that so far as I can ascertain, this is the largest growth from diseased bone that has been brought to the attention of the profession.

Its weight I have estimated to have been at least *one hundred and twenty-four pounds*, and this estimate is based upon the knowledge of the weight of the patient in health, one hundred and nineteen pounds, and when the tumor measured forty inches, one hundred and eighty-nine pounds, an increase of seventy pounds at that time. A further increase of growth of ten inches in circumference adds one-fifth in bulk, and weight fourteen pounds. The general emaciation of body usually amounts to between three and four ninths of the original weight, which would be from forty to fifty pounds. These sums, seventy, fourteen, and forty at least, aggregate a weight of one hundred and twenty-four pounds. This morbid growth, then, attained a weight greater than that of the healthy man himself.

OUTLINE DRAWING SHOWING TUMOR AT DEATH.—Fifty-eight inches in circumferance. Two years and two months of active growth.

I have now given the history and course of this case as they have come under my observation. I wish now to show a heredity that had much to do with the remarkable development just considered. On the mother's side I find nothing relative to any heredity, all having lived to good old age. On the father's side I find that all the ancestors were short-lived people, very few living more than fifty years. The father belonged to a family line, in the mining regions of Dolheim, Prussia, that had intermarried for several generations. He was feeble when born, and was seven years of age before he could walk ; and as he grew to manhood, he was unable to do vigorous work, and was exempted from the required term of service in the Prussian army. His joints were large, loose and clumsy, and the protuberances of the bones unusually prominent. A brother and sister had similar peculiarly-formed joints. He was the father of twelve children, and in them is shown in a remarkable way the heritage transmitted through a diseased parent:

C—, first child, dead, had enlarged ends of tibia and femur at knee-joints.

M—, second child, dead, had large broad knees, with loose articulations.

J—, third child, dead, constitutionally feeble.

J—, fourth child, living, has flat, broad knee, condyles very prominent.

C—, fifth child, living, has an exostosis one and one-half inches in diameter on outside of right femur.

G—, sixth child, the subject of this paper. In addition to the tumor described, there was an exostosis two inches in diameter projecting an inch above the surface, on the inner side of right tibia, as shown in the photograph.

L—, seventh child, living, has a sesamoid bone on the inner side of left knee, apparently in the tendon of the sartorius muscle as it passes behind the inner condyle of the femur. General health feeble.

M—, eighth child, living, has a sesamoid bone near left ankle-joint.

M—, ninth child, living, natural.

S—, tenth child, living, has an unnatural articulation at fifth metacarpo phalangeal joint of left hand.

J— B—, eleventh child, living, natural.

P—, twelfth child, living, natural.

It will be seen, therefore, from this list that nine of the twelve children have shown visible evidence of heredity in enlarged or malformed bones. Nor is this all ; a child, now dead, born to C——, the fifth child, had congenital exostosis on the shaft of the right ulna. And the peculiarly formed knees, common to so many of the family, are observed in a second child now living. This is evidence of heredity in three successive generations to my knowledge.

It is not probable, however, that this hereditary predisposition to localize deposit of bone material has to do with the malignancy of the growth described, but it has much to do with its unusual size and the formation of the bony structure within it, conjointly with the progress of the more active cancerous affection.

The lower extremity of the bone, as shown in the photograph, is more than twice as large as a natural femur at the same point. This is partly due to hypertrophy from disease; but in addition it represents the peculiar form and shape of the extremities of the bones at the joints referred to when tracing the family history. To sum up the interesting points of this case, we may notice:

1st. The existence of an exostosis excited to active growth by violent exercise, augmented by a hereditary tendency to deposit bone material, and finally degenerating into a condition of malignancy.

2d. The site of disease. Osseous tumors of the femur generally appear on the inner side at the lower extremity of the bone. This had a directly opposite location, appearing on the outer side at the upper extremity.

3d. Rapid growth; which in two and one-half years attained a size measuring fifty-eight inches (nearly five feet) in circumference, and a weight of one hundred and twenty-four pounds; and

4th. The awakening of a heredity shown in three successive generations, that in this case was roused to action under proper cause and conditions.

Besides the regular physicians of Racine, Dr. E. B. Wolcott, of Milwaukee, saw the tumor when in an advanced stage of development.

---

IN order to relieve hiccough, inflate the lungs as fully as possible, and thus press steadily and firmly upon the agitated diaphragm. In a few seconds, the spasmodic action of that muscle will cease.

## Figures and Tables

**Figure f1:**
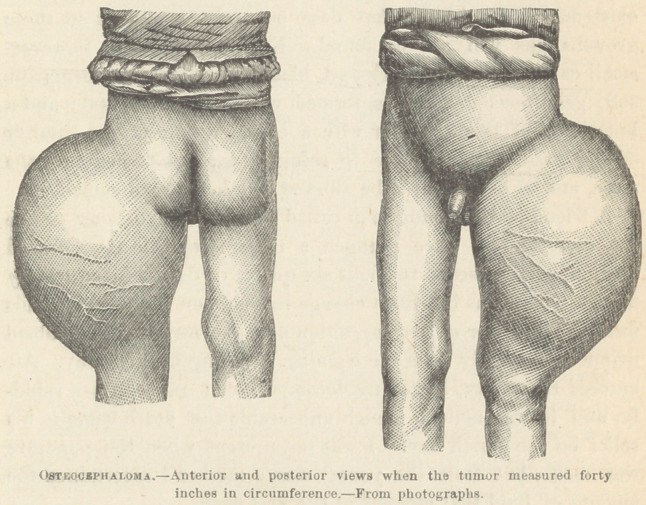


**Figure f2:**
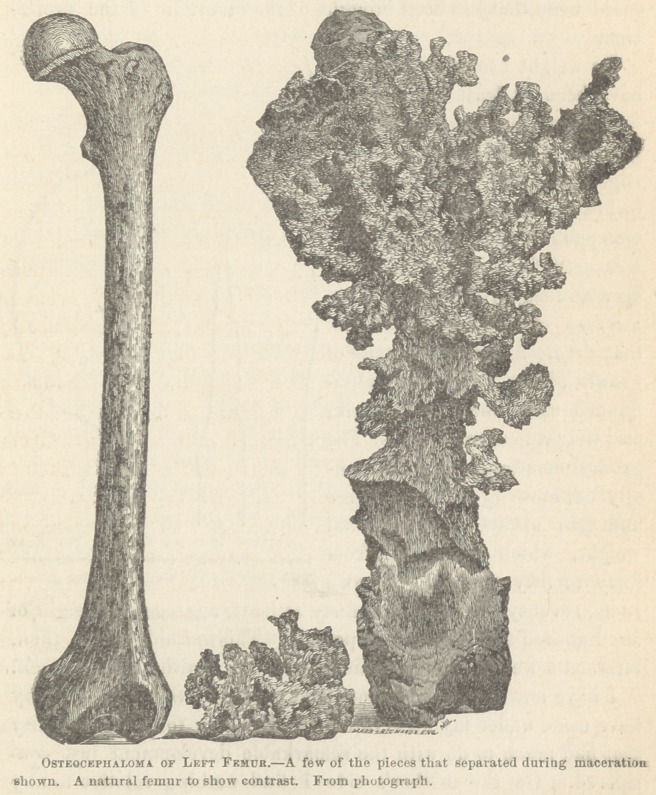


**Figure f3:**